# Sulfoxaflor and Natural Pyrethrin with Piperonyl Butoxide Are Effective Alternatives to Neonicotinoids against Juveniles of *Philaenus spumarius*, the European Vector of *Xylella fastidiosa*

**DOI:** 10.3390/insects10080225

**Published:** 2019-07-30

**Authors:** Beatriz Dáder, Elisa Viñuela, Aránzazu Moreno, María Plaza, Elisa Garzo, Pedro del Estal, Alberto Fereres

**Affiliations:** 1 Escuela Técnica Superior de Ingeniería Agronómica, Alimentaria y de Biosistemas (ETSIAAB), Universidad Politécnica de Madrid (UPM), Avenida Puerta de Hierro 2-4, 28040 Madrid, Spain; 2 Unidad Asociada “Control de Insectos Vectores bajo sistemas de Agricultura Sostenible (IVAS), 28040 Madrid, Spain; 3 Instituto de Ciencias Agrarias (ICA), Consejo Superior de Investigaciones Científicas (CSIC), Calle Serrano 115 dpdo., 28006 Madrid, Spain

**Keywords:** Aphrophoridae, chemical control, Olive Quick Decline Syndrome, emerging diseases

## Abstract

The threat imposed by the bacterial pathogen *Xylella fastidiosa* to crops of utter importance to European agriculture such as olive, stone fruit and grapevine calls for immediate research against the meadow spittlebug, *Philaenus spumarius* (L.), the main European vector. Management tools should consider reducing juveniles of vector populations growing on weeds or cover crops during spring as nymphs have limited movement and do not contribute to disease spread. We examined a wide range of insecticides with different modes of action against *P. spumarius* nymphs in laboratory and semi-field glasshouse conditions. Pyrethroids (delthamethrin and λ-cyhalothrin) and natural pyrethrin (Pirecris^®^) + piperonyl butoxide (PBO) efficacy surpassed 86% after 24 h of exposure, without significant differences in the PBO amount tested. The inclusion of PBO caused a 3-fold increase in the mortality of *P. spumarius* nymphs compared to pyrethrin alone. Sulfoxaflor (Closer^®^) exhibited similar efficacy at 48 and 72 h but it was slow acting and mortality only reached 60% at 24 h. The LC_90_ was 34 ppm at 72 h. Pymetrozine, spirotetramat, azadirachtin and kaolin were not effective against nymphs (mortality <33%) although in azadirachtin-treated plants, mortality had a 3-fold increase from 24 to 72 h. Our results will help decision-making policy bodies to set up a sustainable integrated pest management of *P. spumarius* in areas where *X. fastidiosa* becomes a problem.

## 1. Introduction

*Xylella fastidiosa* (Xanthomonadaceae) Wells et al. [1] is a gram-negative vector-transmitted bacterial pathogen that can affect more than 309 plant species of 63 families [2]. Until now, four subspecies have been described with different host specificity [3]. It is one of the most aggressive pests worldwide, associated with important diseases in a wide range of plants [4,5] and it can sometimes inhabit the plant without causing symptoms [6]. The first syndrome of this bacterial disease was described in 1887 in grapevines in USA, named as Pierce’s Disease (PD), and it has constituted one of the major threats and limiting factors of grapevine production [7]. In 1987, *X. fastidiosa* was associated in Brazil to *Citrus Variegated Chlorosis* (CVC) in citrus and then diagnosed in coffee and ornamental plants in many other American countries (e.g., Brazil, Argentina, Paraguay, Costa Rica, Honduras, Mexico, Venezuela and Ecuador) [8]. Nowadays, the bacterium has been cited in 33 countries on three continents [9].

The impact of *X. fastidiosa*-related diseases is very devastating, not only in agriculture but in the environment as well, because the organism can severely modify the landscape, which in turn has a huge negative effect on the communities living in these areas [10]. Therefore, in the European Union (EU), it is one of the regulated organisms listed in the Council Directive 2000/29/EC on “Protected measures against introduction and spread of harmful organisms in the EU”. However, despite the EU contingency plan [2], the bacterium emerged in Europe in 2013 (*X. fastidiosa* spp. pauca), with at least one million olive trees already wiped out in Southeastern Italy due to the *Olive Quick Decline Syndrome* (OQDS) [11]. In France in 2015, the disease (*X. fastidiosa* ssp. multiplex) was detected on the evergreen ornamental shrub *Polygala myrtifolia* L. and then in the Balearic Islands (Spain) in 2016 (*X. fastidosa* spp. fastidiosa) on this shrub and on cherry trees [12]. At present, *X. fastidiosa* threatens several EU countries and crops of utter importance to European economy such as olive, stone fruit and grapevine [11]. Its dissemination seems unstoppable and in Spain, it has already spread to different mainland areas, mainly attacking almond trees and several ornamental and non-cultivated plants [13]. 

Thus, there is an urgent need to put in place measures to prevent the spread of the disease by avoiding the movement of infected plant material and developing effective strategies against their insect vectors. *Xylella fastidiosa* spread is mediated by numerous species of Auchenorrhyncha Cicadomorpha (superfamilies Cicadellidae, Cercopoidea and Cicadoidea), all of them xylem sap-sucking and widely distributed in temperate regions [14]. In the American continent, the most important vectors are sharpshooter leafhoppers (Cicadellidae, Cicadellinae). Insect vectors of PD in the American continent include *Graphocephala atropunctata* (Signoret), *Draeculacephala minerva* (Ball), *Xyphon (Carneocephala) fulgida* (Nottingham) and the exotic *Homalodisca vitripennis* (Germar) [6,14,15]; for the CVC, *Acrogonia citrina* (Marucci and Cavichioli), *Bucephalogonia xanthophis* (Berg), *Dilobopterus costalimai* (Young), *Macugonalia leucomelas* (Walker) and *Oncometopia fascialis* (Signoret) [16]. In Europe, the predominant species that transmits the bacterium is the spittlebug (Aphrophoridae) *Philaenus spumarius* (L.), polypohagous and univoltine, present from Lapland to the Mediterranean and widely distributed as well in most of the temperate regions of the world [17]. *Neophilaenus campestris* (Fallén) as well as other Cercopoidea and Cicadellinae members are potential vectors of the disease as well [3,17,18,19,20,21]. All these vectors develop in Europe in different herbaceous plants where the nymphs produce spittle-mixing substances released by the anus and some abdominal glands [17,22]. In Spain, juveniles of *P. spumarius* are mainly associated with Asteraceae and those of *N. campestris* with Poacea hosts [13].

Effective management tools to reduce Cercopoidea populations are scarce because they have never been considered pests causing direct damage on crops of economic importance. Nymphs of spittlebugs grow on the natural herbaceous vegetation and have limited mobility [17]. Adults are the only stage contributing to disease spread to woody hosts and there is very limited time frame for prevention because the spittlebugs can acquire and transmit the pathogen in a matter of minutes [23], even though the transmission efficiency increases with plant access time [24]. Reducing juvenile populations by various ways (mechanical or chemical strategies) is therefore of paramount importance to reduce the density of potential vectors of the disease. However, the many non-host plant species identified recently in Italy, in areas where the bacterium is present, opens the possibility of using green barriers for limiting vector movement [25].

Biological control is very limited, although *Gonatocerus ashmeadi* Girault (Hymenoptera: Mymaridae), some species of Trichogrammatidae (Hymenoptera), Coccinellidae and Chrysopidae larvae are known to successfully attack eggs [26,27,28]. Different species of hemipterans, mantises, wasps or spiders have also been cited as preying on nymphs and adults [28]. Harper and Whittaker [29] identified *Ptrerostichus (Platysma) niger* (Schaller) (=*Feronia nigra*) (Carabidae) predating on adults and Nabidae on nymphs in radiolabelled studies. Adults of *P. spumarius* can be attacked as well by the endoparasitoid *Verrallia aucta* (Fallén) (Diptera: Pipinculidae), which is present in Spain [30], and by entomopathogenic fungi of the genus *Entomophtora* sp. [31]. However, the impact of the natural enemies on the populations of the vector seems to be low because they are neither very specific nor very abundant in the areas where the vector develops.

Pesticide development is also scarce because the Cercopoidea species are not pests or are pests of only low-profitable pastures, in which the use of pesticides is unusual. Insecticide applications to the woody target hosts are of limited value to avoid the transmission of *X. fastidiosa* because adults seem to be transient on trees from drying ground vegetation [10]. Therefore, chemical control should mainly focus on nymphs, which have very limited dispersal ability. So far, some synthetic pyrethroids (deltamethrin and λ-cyhalothrin) and neonicotinoids (imidacloprid, acetamiprid and thiamethoxam) have shown good control of adults of *P. spumarius* [32,33]. The naturally derived pyrethrins, organophosphates or essential oils are however much less effective [33]. Kaolin clay could also be an alternative because it increases mortality of *H. vitripennis* and decreases the incidence of Pierce’s Disease in vineyards [15]. Where nymphs are concerned, imidacloprid and deltamethrin can reduce their numbers [34] but there is a lack of information on other pesticide candidates. Moreover, following the recent banning of neonicotinoids imidacloprid, clothianidin and thiamethoxam for outdoor use (EU commission implementing regulations 2018/783, 784 and 785) [35], the number of pesticides to control sap-sucking insects in the EU is now much more limited. As a consequence, there is an urgent need to search for other effective chemical control options.

Thus, the goal of the present work was to investigate the efficacy of chemical alternatives to manage *P. spumarius*, the main European vector of *X. fastidiosa*. A wide range of insecticides targeting sucking insects with different modes of action [36], both authorised and unauthorised in Spain when the test was performed, were tested against juveniles developing on the herbaceous Asteraceace *Sonchus oleraceus* L., one of the most preferred host plant in this area, under laboratory and semi-field glasshouse conditions.

## 2. Materials and Methods 

### 2.1. Insects and Plants

Experiments were conducted at the Institute of Agricultural Sciences of the Spanish National Research Council (ICA-CSIC, Madrid, Spain) (40°26′23″ N, 3°41′14″ W) from April to June in 2018 and 2019. Seeds of *S. oleraceus* were sown with a 1:2 mixture of vermiculite (Asfaltex S.A., Barcelona, Spain) and soil substrate (GoV4, Jiffy International, AS, Norway). Each plant was watered and fertilized with 20-20-20 (N-P-K) Nutrichem (Miller Chemical & Fertilizer Corp., Hanover, PA, USA) at a dose of 1 g L^−1^.

*Philaenus spumarius* nymphs were collected in Sevilla, Huelva and Madrid (Spain) from April to June in 2018 and 2019 on herbaceous plants belonging to families Asteraceae (*Carduus tenuiflorus* Curtis, *Scolymus hispanicus* L.) and Umbelliferae (*Eryngium campestre* L.) and transported to the lab on small *S. oleareceus* potted plants. The mass rearing indoors of this monovoltine vector species is difficult and up to now, it is not possible to have a continuous supply of individuals for testing even though recently some achievements have been reached [37]. Therefore, nymphs collected in the field each week were used right away for the pesticide screening, a number of treatments were performed simultaneously and replicates varied with nymph availability.

### 2.2. Pesticide Screening in Laboratory Conditions

Experiments were conducted inside a climate chamber under controlled conditions at 23:18 °C (day:night) temperature, 16L:8D photoperiod and 60–70% RH. Each experimental unit consisted of ten fourth-instar nymphs transferred with a paintbrush to each 4-true-leaf *S. oleraceus* (1-month-old) grown on a 12 cm diameter pot. Pots were isolated with a plastic and mesh cylinder. After 24 h, the number of nymphs was checked to ensure that there had not been mortality. At this moment, and similarly to natural field conditions, they had already produced consistent spittle to protect them and therefore, plants were sprayed to run off in the lab with the pesticides using a hand sprayer (Matabi Berry^®^ 1.5 L, Goizper Group, Gipuzkoa, Spain). Treatments were performed simultaneously. Roughly, plants received 40 mL solution each. Mortality was evaluated at 24, 48 and 72 h and spittle formation at 72 h. The number of survivors was corrected using Abbott’s formula [38].

Two experiments were set up. Initially, we were interested in establishing the potential of the active ingredient sulfoxaflor (Closer^®^, Dow Agrosciences S.A., Madrid, Spain), authorised in Spain for the control of different sucking insects [39], by establishing the LC_50_ and LC_90_ for vector nymphs. Sulfoxaflor, a sulfoximine pesticide, acts as a modulator of the nicotinic acetylcholine receptors [36]. Increasing concentrations (0, 4, 8, 16, 32 and 64 ppm a.i.) were tested, following the set up described above (n = 4). In the second experiment, we selected eight commercial pesticides that could control nymphs of *P. spumarius* based on the literature, either synthetic or of natural origin and with different modes of action [36] (Table 1). Because there was not any pesticide registered for the control of vectors of *X. fastidiosa* in Spain, Pirecris^®^ was exceptionally authorised by the Spanish Ministry of Agriculture for six months, but it only showed a moderate toxicity against *P. spumarius* nymphs. We decided then to test it together with PBO, a well-known synergist of pyrethrins [40] used in some other authorised Spanish formulations of pyrethrins [41]. For sulfoxaflor, we selected as concentration the LC_90_ at 72 h previously calculated in the first experiment. Pesticides were tested at the maximum field recommended concentration (MFRC) of commercial products in Spain for *Xylella* vectors or for pests with similar feeding behavior in the affected crops (olive tree, almond or grapevine) if non-registered. Two water-mock controls were also included for each pesticide replicate in every experiment because of the overall difficulty in vector management once taken away from its natural habitat and, in the second, PBO was also tested alone at the two selected concentrations (Table 1). 

### 2.3. Greenhouse Experiments under Semi-Field Conditions

Because sulfoxaflor had caused high vector mortality in the lab experiments, we also conducted assays under glasshouse conditions (24:18 ± 2 °C day:night temperature; 14L:10D photoperiod; 60–70% RH). Glasshouse dimensions were 6.4 × 6 × 4.5 m (L × W × H) and light transmission properties of the outer surface were average 50% PAR, 15% UV-A and 10% UV-B. We selected the two concentrations that were the most effective at 72 h (32 and 64 ppm a.i.) when the probit regression was calculated in the lab. Each replicate consisted of four 4-true-leaf *S. oleraceus* (1-month-old) plants that were enclosed in a 50 × 50 cm insect-proof cage with 680 µm mesh (Entomopraxis, Barcelona, Spain), with a soil layer on the bottom of the cage to mimic field conditions and prevent nymphs from falling off the pots. Ten *P. spumarius* nymphs were transferred with a paintbrush to each of the four *S. oleraceus* plants inside the cage. The number of replicates (cages) per treatment was 6 (n = 6, 4 × 10 insects per cage, 240 insects per treatment). Twenty-four hours after insect release, nymphs had already produced consistent spittle so the plants were sprayed to run off point using hand sprayers with the chosen treatment: Sulfoxaflor at 32 and 64 ppm a.i., delthamethrin at 12.5 ppm as positive standard and a water-mock control. Spittle formation, surviving insects settled on plants or walking inside the cage, total surviving insects inside the cage and adult development were evaluated at 24, 48 and 72 h after insect release. 

### 2.4. Statistics

Probit regression of sulfoxaflor was analysed with Polo Plus probit (*p* ≤ 0.05) after assessing fit and overdispersion with other distributions such as logit, which did not provide a better fit than probit. Statistical analyses of pesticide screening on the lab and on the glasshouse were analysed with IBM Statistics SPSS v.23.0 package for Mac (IBM Co., New York, USA) using ANOVA analysis with post hoc LSD or non-parametric Kruskal–Wallis H test when raw or transformed data to logistic regression did not follow criteria for parametric methods (*p* ≤ 0.05) [42]. 

## 3. Results

### 3.1. Sulfoxaflor Efficacy under Laboratory Conditions

**Figure 1:**
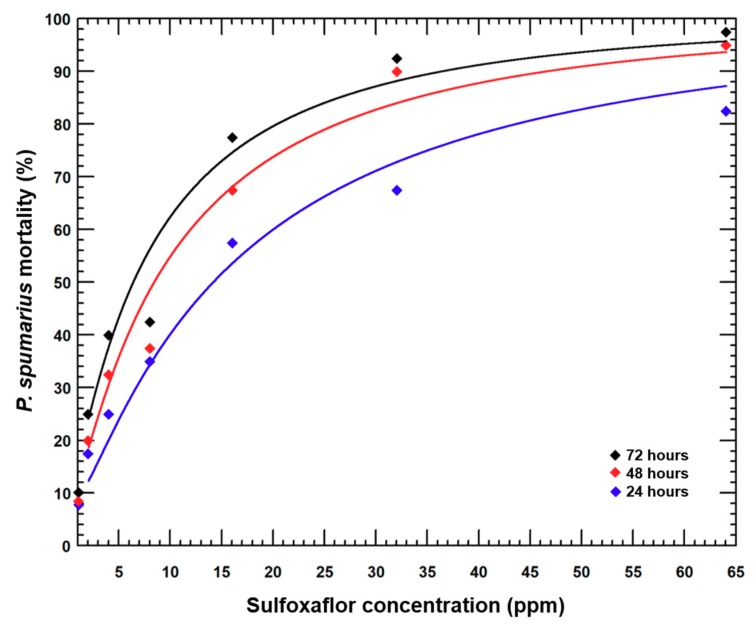
Probit regressions of mortality (%) of *P. spumarius* nymphs after 24, 48 and 72-h exposure in the lab to *S. oleraceus* plants treated with sulfoxaflor.

The two most effective concentrations of sulfoxaflor for *P. spumarius* nymphs at 72 h were 32 and 64 ppm (Figure 1). Nymphal mortality reached 90% only after 48 h of exposure to treated plants at the highest concentration (64 ppm). Control mortality ranged from 7.5 ± 2.5% after 24 h to 10.0 ± 4.0% at 72 h (Figure 1). At 24 h, the LC_50_ was 16.1 ppm and the LC_90_ was 124.0 ppm (χ^2^ = 0.50, df = 4, Het = 0.13, slope ± S.E. = 1.45 ± 0.25, intercept ± S.E. = −1.75 ± 0.35). At 48 h, the LC_50_ was 10.1 ppm and the LC_90_ 43.9 ppm (χ^2^ = 3.05, df = 4, Het = 0.76, slope ± S.E. = 2.00 ± 0.33, intercept ± S.E. = −2.01 ± 0.44). At 72 h, the LC_50_ was 8.2 ppm and the LC_90_ 34.3 ppm (χ^2^ = 3.92, df = 4, Het = 0.98, slope ± S.E. = 2.06 ± 0.35, intercept ± S.E. = −1.88 ± 0.44). 

### 3.2. Sulfoxaflor Efficacy under Glasshouse Conditions

In the control, we observed 75% surviving *P. spumarius* nymphs of the total number released after 72 h, a very good rate given the high mortality of these insects on indoor conditions (Table 2). There was a very good control of *P. spumarius* nymphs when using sulfoxaflor at the highest concentration previously applied in the lab (64 ppm). No adults emerged during the lapse of the experiment and surviving insects after 72 h were statistically similar to those registered in the positive standard deltamethrin at a concentration of 12.5 ppm. Sulfoxaflor at 32 ppm gave a poorer control, because the number of nymphs that survived was higher throughout the experiment (Table 2). 

### 3.3. Pesticide Screening under Laboratory Conditions 

Table 3 shows mortality of juvenile *P. spumarius* with active ingredients arranged from most to least successful at 72 h (Table 3). Mortality on water-mock control only reached 5.5 ± 1.2% at 72 h. PBO at the two concentrations applied caused low mortality (under 17.5% at 24 h and under 26.7% at 72 h), similar to other products tested (e.g., spirotetramat, pymetrozine, pyrethrin). Pyrethroids (λ-cyhalothrin and delthamethrin), sulfoxaflor and pyrethrin supplemented with synergist piperonyl butoxide (PBO) caused high mortality to *P. spumarius* nymphs. Excellent control was achieved by natural pyrethrin supplemented with PBO and there were no significant differences between the two concentrations tested (1% or 3%). In contrast, the mortality under natural pyrethrin alone was much lower (under 33% at 72 h). Pyrethrin and PBO showed synergy because the mortality caused by either alone was at least 3-fold lower than that caused when applied together. There were no significant differences among pyrethrin supplemented with PBO and λ-cyhalothrin at any evaluation time. Mortality hardly varied on pyrethroid-treated plants from 24 to 48 or 72 h, proving the rapid knockdown response that is typical for this group of insecticides. Sulfoxaflor action was slower, and initially the mortality recorded at 24 h only reached 61.7 ± 5.8%. However, at 72 h, mortality increased up to 82.5%, being significantly equal to that of deltamethrin. Remaining pesticides (pymetrozine, spirotetramat, azadirachtin and kaolin) did not cause more than 33% mortality at 72 h (Table 3). In the azadirachtin treatment, mortality increased from 7.5 ± 3.0% at 24 h to 25.0 ± 6.3% at 72 h. With regard to spittle formation, the results were cohesive with mortality and nymphs were able to develop spittle only on plants treated with insecticides that caused low mortality (natural pyrethrin, PBO, pymetrozine, spirotetramat and azadirachtine) or in sulfoxaflor, initially slow acting (61.7% mortality at 24 h) (Table 3).

## 4. Discussion

The threat imposed by *Xylella fastidiosa* to agriculture calls for immediate research on management tools against the meadow spittlebug, *Philaenus spumarius*, the main European vector of *X. fastidiosa.* Chemical management actions for a containment strategy against this bacterial disease should focus on nymphs, because they have limited movement ability and do not contribute to disease spread [17]. Adults of *P. spumarius*, on the contrary, are extremely polyphagous and transient vectors that contribute to the secondary spread of *X. fastidiosa* when moving from tree to tree, despite the fact that they are non-infective when emerging [23]. Moreover, the bacterial transmission is a fast process because it is non-circulative and propagative without a latent period [6,23]. Therefore, chemical control against the adult stage has limited value to contain the disease. Neonicotinoid insecticides are effective for nymph and adult sharpshooter control [6]. However, in Europe, the outdoors use of imidacloprid and other products of this group have been banned recently [35] and other alternatives must be explored.

In the present work, we report the results of pesticide efficacy assays under laboratory and semi-field glasshouse conditions, with nymphs collected from host plants in the field and transferred onto a different host (*S. oleraceus*) for the experiments. This manipulation can be stressful or lethal, as we have observed over the course of experimentation with this insect. However, we managed to keep mortality below 7% on water-mock controls in laboratory conditions and below 25% in semi-field conditions. These rates are acceptable for this insect due to its high dependence on humidity and the overall difficulty in its management once taken away from its natural habitat. 

Pyrethroids are effective against sharpshooter adults and nymphs [32,33,34,44] and they are usually included in conventional management practices against *H. vitripennis*, a vector of PD on vineyards in the American continent [32]. As expected, in our laboratory tests, the two tested pyrethroids (delthamethrin and λ-cyhalothrin) offered a good control of *P. spumarius* nymphs and were fast acting. Delthamethrin and λ-cyhalothrin exhibited high efficacy 24 h after exposure (mortalities >86.8%) and this efficacy was maintained after 72 h. In contrast, other synthetic pesticides tested—pymetrozine (ion channel modulator) [36] and spirotetramat (inhibitor of acetyl CoA carboxylase) [36]—caused a low mortality of nymphs (under 20% at 24 h and 30% at 72 h), which is in in accordance with the results of Dongiovanni et al. [34]. Sulfoxaflor (Closer^®^), applied at 64 ppm under glasshouse conditions, exhibited an efficacy very similar to deltamethrin, with 95% reduction of insects on plants compared to controls at 24 h after exposure and the absence of emerged adults. Applied at 32 ppm, the efficacy slightly decreased (87% reduction at 24 h and 1.3 ± 0.5 adults emerged after 72 h). 

Since the directive 2009/128/EC of the European Union encouraged more environmentally friendly pesticides, natural products which harbor a huge diversity of substances [45] are considered a good alternative to traditional pesticides in crop protection [46]. Natural pyrethrins are one of the most commonly used pesticides because of the quick knock-down effect but they can also be swiftly detoxified by insect enzymes, thus requiring, in general, synergists to prolong their action (PBO being one of the most used). Natural pyrethrins have been reported to fail in controlling *P. spumarius* populations, with only 25% mortality after 72 h [34]. Under our conditions, Pirecris^®^ raised mortality up to 32%, which is still insufficient. However, when PBO was added, the mortality of nymphs at 24 h surpassed 95% irrespective of the synergist amount (1% or 3%), it did not decrease at 72 h and was not significantly different from that of λ-cyhalothrin. Currently, there is not any insecticide authorised in Spain containing PBO against *P. spumarius*—only pyrethrin alone—however, we believe it should be considered because of its potential to enhance pyrethrin efficacy. 

The other natural products tested—azadirachtin (uncertain mode of action) [36] and kaolin (obstruct insect movement and feeding) [47]—caused low mortality to *P. spumarius* nymphs, which agree with results of Dongiovanni et al. [34,41]. Mortality of *P. spumarius* exposed to both products was under 26% at 72 h and in azadirachtin-treated plants had a 3-fold increase from 24 (7.5%) to 72 h (25%). Kaolin, a repellent formulation of aluminum silicate, has been, however, successfully applied against *H. vitripennis* in vineyards affected by Pierce’s disease [15,19]. As the overall effect of kaolin is to create a particle film that disrupts vector orientation, landing and feeding behavior [48], it could potentially have contributed to the impairment of spittle formation. In order to mimic field conditions in our trials, nymphs were transferred to plants 24 h prior spray application to allow them to start creating spittle because nymphs cannot survive outside the spittle [49]. In the case of kaolin-treated plants, this process was not affected (spittle was present in 67% of the plants compared to 76% in the control). The spittle formation was only severely decreased or blocked in those insects exposed to quick acting pesticides (pyrethroids and natural pyrethrin + PBO). The spittle is probably good protection against the penetration of pesticides because in pymetrozine, spirotetramat, azadirachtin and natural pyrethrin, the number of plants showing spittle at the end of the experiment was high (50–77%) and the nymphal mortality was low (under 24% at 24 h and 33% at 72 h). However, there must be more factors involved in spittle disruption because in sulfoxaflor, the number of spittle had a 1.3-fold reduction compared to controls but nymphal mortality reached 61.7% at 24 h and 82.5% at 72 h after exposure. Therefore, based in our results, neither azadirachtin or kaolin were a good alternative to control *P. spumarius* nymphs under our experimental conditions even though both are authorised in Spain against *P. spumarius* [50].

Our screening of commercial products under laboratory and semi-field conditions constitutes the first step to ascertain the efficacy of a wide range of insecticides, with different modes of action, for the control of *P. spumarius*. These preliminary results have allowed the selection of two promising insecticides to be tested under more realistic conditions: Sulfoxaflor and natural pyrethrin supplemented with PBO. Both products are viable alternatives to the pyrethroids authorised in Spain at present for the chemical control of the main vector of *X. fastidiosa* and deserve further research.

## 5. Conclusions

As a whole, the pyrethroids deltamethrin and λ-cyhalothrin (as already known), and also sulfoxaflor (Closer^®^) and the naturally-derived pyrethrin Pirecris^®^ + PBO, were successful in controlling *P. spumarius* nymphs under laboratory conditions. Natural pyrethrin can be a good alternative to traditional pesticides since the EU encourages more environmentally friendly products. However, when it was applied alone, nymphal mortality suffered a 3-fold decrease. The synergist PBO prolongs its action and enhances its efficacy, being non-toxic alone regardless of the concentration used in our assay (1–3%) (mortality <27% at 72 h). In contrast, pymetrozine, spirotetramat, azadirachtin and kaolin were not effective. Given the need of knowledge for *X. fastidiosa* containment measurements in Europe, one of the most serious threats to our agriculture, our research on the chemical control of the main European vector offers interesting alternatives to be implemented in regions where *X. fastidiosa* is present. Results presented in this manuscript are preliminary data on the efficacy of insecticides under laboratory and semi-field conditions, which allows the selection of promising candidates to be assessed in field tests.

## Figures and Tables

**Table 1:** Active ingredients tested in laboratory experiments against *P. spumarius* nymphs. Commercial products, mode of action according to IRAC classification, authorisation in EU [43], commercial product concentrations and replicates for each treatment are detailed.

Active Ingredient	Commercial Product	Company	IRAC	Authorized	MFRC ^#^ Commercial Product	Replicates *
Water-mock	-	-	-	-	-	29
1% PBO ^†^	Piperonyl butoxide 90%	Alfa Aesar (Lancashire, United Kingdom)	-	No	10 mL/L	12
3% PBO	Piperonyl butoxide 90%	Alfa Aesar (Lancashire, United Kingdom)	-	No	30 mL/L	12
Kaolin	Surround^®^ 95% [WP] P/P	Tessenderlo (Overpelt, Belgium)	-	Yes	50 g/L	12
Azadirachtin	Align^®^ 3.2% [EC] P/V	Sipcam Inagra (Valencia, Spain)	UN ^‡^	Yes	1.5 mL/L	12
Delthamethrin	Decis Evo^®^ 2.5% [EW] P/V	Bayer (Madrid, Spain)	3A	Yes	0.5 mL/L	12
λ-cyhalothrin	Karate Zeon^®^ 10% [CS] P/V	Syngenta (Madrid, Spain)	3A	Yes	0.2 mL/L	12
Pyrethrin	Pirecris^®^ α 2% [EC] P/V	Seipasa (Valencia, Spain)	3A	Yes	1.5 mL/L	13
Pyrethrin + 1% PBO	Pirecris^®^ 2% [EC] P/V	Seipasa (Valencia, Spain)	3A	No	1.5 + 10 mL/L	12
Pyrethrin + 3% PBO	Pirecris^®^ 2% [EC] P/V	Seipasa (Valencia, Spain)	3A	No	1.5 + 30 mL/L	12
Sulfoxaflor	Closer^®^ [SC] P/V	Dow Agrosciences (Madrid, Spain)	4C	Yes	0.24 mL/L	12
Pymetrozine	Plenum^®^ 50% [WG] P/P	Syngenta (Madrid, Spain)	9	Yes	0.5 g/L	12
Spirotetramat	Movento^®^ 150 O-TEQ 15% [OD] P/V	Bayer (Madrid, Spain)	23	Yes	0.5 mL/L	12

^†^ PBO: piperonyl butoxide, ^‡^ UN: unknown, ^#^ MFRC: maximum field recommended concentration, * number variable depending on nymph availability (insects collected in the field), α exceptionally authorised in Spain in 2018 for the control of vectors of *X. fastidiosa.*

**Table 2:** Glasshouse experiments. Effect of sulfoxaflor and deltamethrin on *P. spumarius* nymphs (mean ± SEM per cage) after 24, 48 and 72 h-exposure to the insecticides in *S. oleraceus* plants. Different letters within the same column indicate statistical differences according to ANOVA or Kruskal–Wallis H tests (*p* ≤ 0.05).

Treatment	Spittle ^†^	Insects Settled on *S. oleraceus* (%)			Insects Walking inside the Cage (%)			Total Insects Alive inside the Cage (%)			Number of Adults ^#^		
		24 h	48 h	72 h	24 h	48 h	72 h	24 h	48 h	72 h	24 h	48 h	72 h
Water-Mock Control	Yes	78.8 ± 4.9 a	73.3 ± 5.5 a	58.8 ± 7.8 a	8.8 ± 3.3 a	9.6 ± 2.5 a	18.8 ± 9.0 a	87.5 ± 4.2 a	82.9 ± 4.6 a	77.5 ± 5.4 a	6.7 ± 2.2 a	12.3 ± 3.7 a	25.2 ± 2.0 a
Sulfoxaflor 32 ppm	No	12.5 ± 2.2 b	8.8 ± 2.1 b	7.5 ± 2.0 b	0.0 ± 0.0 a	0.0 ± 0.0 b	0.0 ± 0.0 b	12.5 ± 2.2 b	8.8 ± 2.1 b	7.5 ± 2.0 b	0.3 ± 0.6 b	0.3 ± 0.2 b	1.3 ± 0.5 b
Sulfoxaflor 64 ppm	No	3.8 ± 1.5 c	2.5 ± 1.6 bc	1.7 ± 1.2 bc	0.0 ± 0.0 a	0.0 ± 0.0 b	0.0 ± 0.0 b	3.8 ± 1.5 c	2.5 ± 1.6 bc	1.7 ± 1.2 bc	0.0 ± 0.0 b	0.0 ± 0.0 b	0.0 ± 0.0 b
Delthamethrin 12.5 ppm	No	0.0 ± 0.0 c	0.0 ± 0.0 c	0.0 ± 0.0 c	0.0 ± 0.0 a	0.0 ± 0.0 b	0.0 ± 0.0 b	0.0 ± 0.0 c	0.0 ± 0.0 c	0.0 ± 0.0 c	0.0 ± 0.0 b	0.0 ± 0.0 b	0.0 ± 0.0 b
Statistics		F = 19.579 df = 3(20) *p* < 0.001	F = 9.775 df = 3(20) *p* < 0.001	F = 6.019 df = 3(20) *p* = 0.004	F = 8.733 df = 3(20) *p* = 0.001	F = 12.864 df = 3(20) *p* < 0.001	F = 11.307 df = 3(20) *p* < 0.001	H = 20.713 df = 3 *p* < 0.001	F = 13.800 df = 3(20) *p* < 0.001	H = 18.803 df = 3 *p* < 0.001	H = 15.555 df = 3 *p* < 0.001	H = 14.589 df = 3 *p* < 0.001	H = 19.768 df = 3 *p* < 0.001

^†^ Spittle formation after 72 h of insecticide treatment, ^#^ adults molted during the time lapse of the experiment from the L4 nymphs released at the beginning of assay.

**Table 3:** Laboratory experiments. Lethal effect of pesticides on *P. spumarius* nymphs (mean ± SE) after 24, 48 and 72 h-exposure to the insecticides on *S. oleraceous* plants and spittle formation at 72 h. Different letters within the same column indicate statistical differences according to Kruskal–Wallis H test (*p* ≤ 0.05).

Active Ingredient	MFRC ^†^ Commercial Product	Plants with Spittle/Plants Tested at 72 h	Corrected Mortality (%)		
			24 h	48 h	72 h
Pyrethrin + 3% PBO	1.50 + 30 mL/L	0/12 = 0%	96.0 ± 2.6 a	96.0 ± 2.6 a	96.0 ± 2.6 a
Pyrethrin + 1% PBO	1.50 + 10 mL/L	0/12 = 0%	97.5 ± 1.3 a	96.7 ± 1.4 a	95.9 ± 1.9 a
λ-cyhalothrin	0.20 mL/L	0/12 = 0%	96.7 ± 1.4 a	95.0 ± 1.5 a	94.2 ± 1.9 a
Delthamethrin	0.50 mL/L	0/12 = 0%	86.8 ± 2.8 b	86.8 ± 2.5 b	83.5 ± 3.1 b
Sulfoxaflor	0.24 mL/L	7/12 = 58%	61.7 ± 5.8 c	76.7 ± 5.4 c	82.5 ± 3.9 b
Pyrethrin	1.50 mL/L	10/13 = 77%	23.9 ± 6.7 d	24.6 ± 6.6 d	32.3 ± 7.6 c
Pymetrozine	0.50 g/L	8/12 = 67%	15.0 ± 3.6 d	20.9 ± 6.0 d	28.4 ± 5.9 c
1% PBO	10 mL/L	10/12 = 83%	13.3 ± 3.1 d	20.2 ± 4.3 d	26.7 ± 5.0 c
Azadirachtin	1.5 mL/L	6/12 = 50%	7.5 ± 3.0 d	17.5 ± 4.6 d	25.0 ± 6.3 c
3% PBO	30 mL/L	11/12 = 92%	17.5 ± 2.5 d	20.2 ± 0.0 d	22.5 ± 2.5 c
Spirotetramat	0.5 mL/L	8/12 = 67%	15.8 ± 3.6 d	19.2 ± 3.6 d	21.7 ± 4.9 c
Kaolin	50 g/L	8/12 = 67%	12.5 ± 5.7 d	16.7 ± 6.3 d	18.3 ± 6.6 c
Water-mock ^#^	-	16/21 = 76%	4.0 ± 1.1	4.5 ± 1.1	5.5 ± 1.2
Statistics			H = 106.112 df = 11 *p* < 0.001	H = 101.994 df = 11 *p* < 0.001	H = 101.407 df = 11 *p* < 0.001

^†^ MFRC: maximum field recommended concentration, ^#^ uncorrected water-mock data were used to correct mortality in treatments.
